# Unmasking the Determinants of Loss to Follow-Up in Pulmonary Tuberculosis: A Study in Selangor, Malaysia

**DOI:** 10.3390/tropicalmed10080226

**Published:** 2025-08-12

**Authors:** Sumarni Mohd Ghazali, Kee Chee Cheong, Mohamad Nadzmi Md Nadzri, Nur’Ain Mohd Ghazali, Lim Mei Cheng, Lonny Chen Rong Qi Ahmad, Mohd Kamarulariffin Kamarudin, Nur Ar Rabiah Ahmad, Asrul Anuar Zulkifli, Cheong Yoon Ling, Qistina Ruslan, Sarbhan Singh, Balvinder Singh Gill, Asmah Razali, Nuur Hafizah Md Iderus

**Affiliations:** 1 Institute for Medical Research, National Institutes of Health, Ministry of Health, Shah Alam 40170, Malaysia; mohamad.nadzmi@moh.gov.my (M.N.M.N.); nurainmg@moh.gov.my (N.M.G.); dr.limmeicheng@moh.gov.my (L.M.C.); lonny@moh.gov.my (L.C.R.Q.A.); drkamarulariffin@moh.gov.my (M.K.K.); arrabiah@moh.gov.my (N.A.R.A.); asrul.anuar@moh.gov.my (A.A.Z.); cheongyl@moh.gov.my (C.Y.L.); qistina@moh.gov.my (Q.R.); lssarbhan@moh.gov.my (S.S.); drbsgill@moh.gov.my (B.S.G.); nuurhafizah@moh.gov.my (N.H.M.I.); 2 Sector for Biostatistics and Data Repository, National Institutes of Health, Ministry of Health, Shah Alam 40170, Malaysia; kee@moh.gov.my; 3 Disease Control Division, Ministry of Health Malaysia, Putrajaya 62590, Malaysia; dr.asmahrazali@moh.gov.my

**Keywords:** tuberculosis, loss to follow-up, interrupted treatment, treatment compliance

## Abstract

Adherence to the 6-month tuberculosis (TB) treatment regimen is challenging due to its duration and side effects, with various factors influencing patient compliance. A retrospective cross-sectional study was conducted among newly diagnosed pulmonary TB (pTB) patients in Selangor, Malaysia, undergoing treatment in government primary care clinics and hospitals. Patients who were lost to follow-up (LTFU) within the first six months were determined by reviewing patient records and the national TB registry. Logistic regression analysis identified sociodemographic and clinical factors associated with LTFU. Of the 699 pTB patients, 55 (7.9%) were lost to follow-up. Factors significantly associated with LTFU included age (higher in 25–44-year-olds, adjusted odds ratio (aOR): 2.83), unmarried status (aOR: 2.17), lower education level (aOR: 6.13), being a smoker (aOR: 2.65), and unawareness of TB diagnosis (aOR: 38.14). A significant interaction was found between education level and awareness of diagnosis, with unawareness having a stronger association with LTFU among higher-educated patients. Young adults, those with a lower education level, unmarried individuals, smokers, and those unaware of their TB diagnosis are at higher risk of LTFU. These factors can be used for rapid risk assessment. Intensive counselling and health education at treatment initiation, particularly for at-risk patients, are crucial for preventing LTFU.

## 1. Introduction

Pulmonary TB (pTB) has afflicted humanity for centuries; it is among the earliest infectious diseases known to man. Though it is preventable and now curable, effective treatment having been available for more than half a century, the tuberculosis pandemic continues to worsen, with most cases occurring in South East Asian countries. Currently classified as an intermediate TB burden country according to WHO, between 2015 and 2021, Malaysia had an 8.4% increase in TB incidence, and a 46% increase in TB deaths [1]. The increased incidence may reflect both a true rise in disease burden and improved detection due to enhanced surveillance and diagnosis; however, it also highlights the importance of addressing factors such as treatment loss to follow-up (LTFU), which can lead to prolonged infectiousness, on-going transmission, and ultimately contribute to further increase in incidence.

TB treatment in Malaysia is primarily provided by the public healthcare system for free for Malaysian citizens, and for non-citizens it is heavily subsidised. Achievement of the End TB strategy goal of a 95% reduction in TB mortality rate by 2035 [2] is contingent upon successful treatment, but the therapeutic success target of 85% among newly diagnosed individuals with a positive smear has largely not been achieved. Among patients who started treatment in 2019, only 55% were successful [1]. The low success rate may be attributed in part to LTFU [3]. The initial two months of treatment is the intensive phase (daily medication), followed by four months in the maintenance phase (thrice weekly medication). Loss to follow-up is said to occur primarily due to the long treatment duration (minimum 6-month course), some within the intensive phase of treatment [4,5], but mostly later [6]. Treatment LTFU has serious consequences, including higher probability of relapse following treatment, emergence of drug-resistant species of *Mycobacterium*, and death of the afflicted patient [7]. An equally grave concern is LTFU patients who are still infectious circulating in the community and posing a risk of TB transmission.

Factors previously associated with treatment LTFU include sociodemoeconomic factors (male gender [4,8]), younger age [9], low income [4], low educational level, [8], low socioeconomic status [7], availability of transportation [7], work interference to TB treatment [9], and distance to nearest healthcare facilities [7]. Lifestyle factors may also play a role, such as alcoholism [4,10] and current smoking status [9].

Loss to follow-up can also be associated with clinical and treatment factors such as daily DOTs [9], previous LTFU [4,8,10], HIV positivity [4,10], other comorbidities [7,8,10], mild and moderate side effects of TB drugs [9], retreatment [9], and clinical symptoms of TB resolving within 2 months [9]. Attitudes and perceptions such as not telling friends about one’s diagnosis of TB [9], knowledge of treatment duration [9], perceived stigma [7], patient’s health beliefs [7], and herbal medication use [4] have also been implicated.

Given the myriad of factors that may affect TB treatment LTFU, the goal of this study is therefore to identify sociodemographic and treatment variables associated with TB treatment LTFU among pulmonary TB patients undergoing treatment in government health facilities in Malaysia that may suggest additional measures that can be considered to reduce treatment LTFU. While previous local studies in Selangor by Sharani et al. [11] and Suliman et al. have investigated TB treatment LTFU, our study differs in key areas. Sharani et al. focused exclusively on TB patients who were smokers, a subgroup predominantly composed of males, thereby limiting the assessment of broader sociodemographic factors. Suliman et al., on the other hand, restricted their analysis to patients from urban districts and examined treatment interruption only during the intensive phase. In this study, we aim to analyse LTFU among patients from both urban and rural settings and over the full course of TB treatment to be more representative of this patient population and capture important contextual disparities in treatment adherence which are often overlooked when studies are confined to urban populations. Including rural communities ensures that our findings are more inclusive and informative for equitable public health planning.

## 2. Materials and Methods

### 2.1. Study Design and Sampling

We conducted a retrospective cross-sectional survey of patients undergoing treatment for pulmonary tuberculosis in all government health clinics and hospitals in the state of Selangor between 1 February 2017 and 31 December 2017, except for facilities that reported having no newly diagnosed pTB cases during this period. Estimation of the minimum sample size required was based the following formula for comparing two proportions by Dupont: *n* = (z_α/2_ + z_β_)^2^ × (p_1_(1 − p_1_) + p_2_(1 − p_2_))/(p_1_ − p_2_)^2^ [12]. Given an alpha value (α) of 0.05, a power (1 − β) of 80%, and an assumed proportion of low education level among LTFU patients of 0.79 (p_1_) and among non-LTFU of 0.72 (p_2_) (Garrido et al., 2010) [10], the minimum sample size amounted to 1252.

### 2.2. Data Collection

Newly diagnosed clinically or bacteriologically confirmed pulmonary TB (pTB) patients who had been on anti-TB treatment for less than one month were identified during scheduled data collection visits at Ministry of Health clinics and hospitals across Selangor and recruited for the study. Retreatment cases (relapse, treatment after failure, and treatment after loss to follow-up) as well as mentally ill and mentally challenged patients were excluded. Written informed consent was obtained from patients prior to data collection, which consisted of a face-to-face interview with the aid of a pre-tested, structured questionnaire (Supplementary File S1). Trained interviewers conducted face-to-face interviews with participants to minimise potential misinterpretation of questions and ensure standardised data collection.

The questionnaire was constructed specifically for this study, except for the knowledge and stigma scales, which were adopted from a study by the World Health Organization Regional Office for the Eastern Mediterranean [13]. These instruments have also been applied in other countries within the WHO Eastern Mediterranean Region, and before deployment in this study, the scales were pre-tested for clarity and cultural appropriateness. The full questionnaire contained questions on patients’ sociodemographic, lifestyle, psychosocial, and treatment experience. Sociodemographic variables consisted of age, gender, education level (no formal education/primary school/secondary school/tertiary), employment status (employed/unemployed), marital status (single/married/divorced/widowed), and nationality (Malaysian/non-Malaysian). Education level was dichotomised into two groups according to the highest level of education attained, i.e., secondary school education or less and tertiary education (college diploma and above). Employment status refers to whether individuals were gainfully employed at the time of the interview. The questionnaire was pre-tested to ensure its comprehensibility and clarity before deployment.

Lifestyle factors consisted of smoking status (current smoker vs. non-current smokers (never or former smoker)) and alcohol consumption (consumed alcoholic drinks in the past 7 days). Psychosocial factors refer to patients’ total treatment delay, TB knowledge, and TB stigma. Total treatment delay (number of days from date of first symptom onset to date of treatment initiation) was determined from patient self-report and treatment records [14]. TB knowledge was assessed using a 7-item questionnaire in which correct answers were given a score of 1 and wrong answers were scored as 0. TB knowledge scores ranged from 0 to 7 (higher scores indicate better knowledge). The respondents were posed the question “What is the name of the disease you are being treated for?” Those who responded with TB or tuberculosis were classified as “Aware of TB diagnosis”, and those who answered otherwise as “Not aware of TB diagnosis”. TB stigma was measured using a 15-item questionnaire adapted from a study by the WHO Eastern Mediterranean Region Office. For the psychosocial variables measured on an ordinal scale (i.e., total treatment delay, TB knowledge, and TB stigma), respondents were classified into two categories (low and high) based on the median values as cut-offs (≤median vs. >median, respectively).

Questions about TB treatment experience were included to capture information on patients’ experiences with side effects of TB drugs, communication with healthcare providers regarding the treatment regimen, satisfaction with the health services provided, and use of supplements. Patients’ treatment LTFU status was determined at the end of the data collection period, through examination of the patients’ DOTs records and case notes, or if both were not available, from records in the TB patient registry. Patients whose treatment was interrupted for at least 2 months within the first six months of TB treatment initiation were classified as LTFU, which is in accordance with the World Health Organization report, which regards interruption of treatment for 2 consecutive months as loss to follow-up (LTFU) [15]. However, whereas the WHO also regards those who did not begin treatment as LTFU, in this study, we only considered patients who have initiated treatment as mentioned. Patients who did not initiate treatment were excluded because our study was conducted in TB treatment clinics, and these individuals were not accessible for recruitment, as they never presented to these facilities to begin treatment. The study protocol was vetted and approved by the Malaysian Ministry of Health Medical Research and Ethics Committee (NMRR-16-978-30978).

### 2.3. Statistical Methods

The proportion of respondents who were LTFU was analysed and described in frequencies and percentages, with further stratification by sociodemographic, lifestyle, psychosocial, and treatment experience variables. Univariable analysis was initially conducted to identify potential risk factors of LTFU. This involved testing associations between LTFU and each sociodemographic, lifestyle/psychosocial, and treatment experience variable using Pearson’s chi-square or Fisher’s exact test, as appropriate. TB knowledge was analysed using the chi-square test for trend.

This was followed by multiple logistic regression modelling with only the significant variables with *p* < 0.25 from the univariable analysis. This was followed by multiple regression with backward stepwise variable selection using LR, which retains only variables significant at *p* < 0.05. Two-way interactions between predictors in the preliminary final model were examined by including the interaction terms in the model, one interaction term at a time as well as assessment of multicollinearity using the variance inflation factor (VIF). The final model was examined for goodness of fit by the Hosmer–Lemeshow chi-square test, percentage of correct classification, and area under ROC curve. All statistical analyses were performed using SPSS version 29 (IBM Corp. Released 2019. IBM SPSS Statistics for Windows, Version 29.0. Armonk, NY, USA, IBM Corp.).

## 3. Results

Patients were recruited from a total of 51 health clinics and 12 hospitals facilities in Selangor with newly diagnosed pTB cases during the study period. At the end of the 6-month follow-up, 699 out of 732 patients initially enrolled in the study were included in the analysis. The 33 cases excluded were patients who died during treatment (*n* = 5), had missing crucial data (*n* = 18), immigrants who were deported to their home country (*n* = 5), or had their diagnoses changed to something other than tuberculosis (*n* = 5).

We conducted post hoc (retrospective) power analysis to assess whether the final sample retained sufficient statistical power to detect a meaningful effect. Although the original target sample size was 1252, a total of 732 patients were enrolled, and 699 were retained in the final analysis. The post hoc power calculation was based on the variable with the smallest observed effect size, i.e., marital status, where 55 were LTFU and 644 were not. Among non-LTFU, 36% were single, divorced, or widowed. Using the observed odds ratio for LTFU of 2.4 (for single/divorced/widowed compared to married), the calculated power to detect a statistically significant effect at α = 0.05 was 87.9%. These findings indicate that, despite the reduced sample size, the final dataset remained adequately powered for detecting meaningful associations.

The sociodemographic characteristics of the study respondents and of LTFU patients are shown in Table 1. Almost a third of the patients (225/699 or 32.2%) had a history of treatment interruption (missed at least one day’s medication), but only 7.9% (55/699) met the criteria for LTFU, i.e., treatment interrupted for more than 2 months consecutively.

Table 1, Table 2 and Table 3 show the findings from univariable analysis of LTFU by sociodemographic (Table 1), lifestyle and psychosocial (Table 2), and treatment experience variables (Table 3). Significant crude associations were found for age, education, employment status, marital status, and smoking status with LTFU.

Table 4 shows the results of multivariable analysis, in which only age, education, marital status, smoking status, and awareness of TB diagnosis remained significant (Table 4). The odds of LTFU were significantly higher among younger (25–44-year-olds) (adjusted odds ratio (aOR) 2.83, 95% CI: 1.40–5.72), unmarried (single/divorced/widowed) (aOR 2.17, 95% CI: 1.14–4.14), and smoking patients (aOR 2.65, 95% CI: 1.43–4.92).

**Figure 1:**
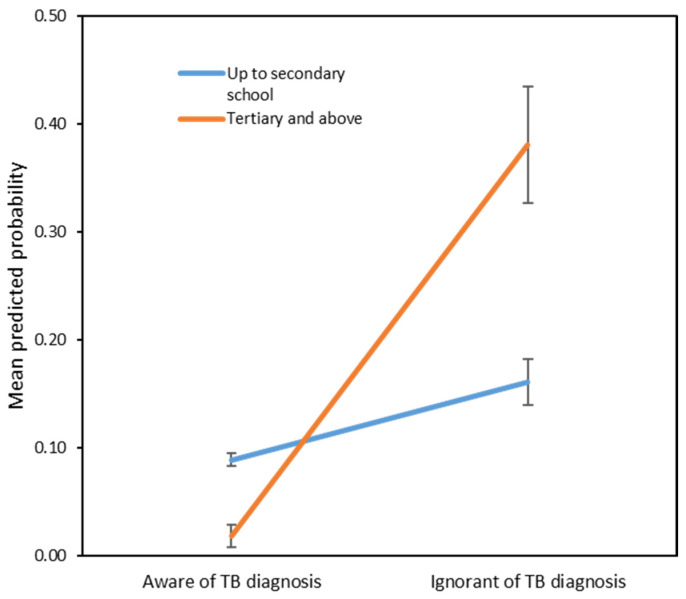
Interaction plot showing the effect of awareness of TB diagnosis on the probability of LTFU by education level. The plot is based on a multivariable logistic regression model. Predicted probabilities were derived from the adjusted model.

There was significant interaction between education level and awareness of TB (*p* = 0.017), indicating that the effect of awareness on the odds of LTFU varies depending on the level of education. Specifically, probability of LTFU was higher among patients with education beyond secondary school (tertiary education), but were not aware of their TB diagnosis, but the probability of LTFU was lower among higher-educated patients if they were aware of their TB diagnosis (Figure 1).

## 4. Discussion

The prevalence of LTFU in our study population was 7.9%. This figure is notably low when compared to rates reported in other countries. For instance, a study in Kuwait found an 11.5% prevalence of LTFU among patients registered for pulmonary tuberculosis treatment between 2010 and 2012 [8]. Interestingly, a more recent study by Suliman et al. [5] conducted in the same state as our study reported a much higher prevalence of 23.7%. However, it is crucial to note that their study focused on treatment interruption (14 days or more in the intensive phase of TB treatment), not treatment LTFU as per the World Health Organization (WHO) definition. It is important to distinguish between these terms. Patients might intermittently miss a few days’ medication but still ultimately complete their full course of treatment. The WHO defines LTFU as a TB patient who either did not start treatment or whose treatment was interrupted for two consecutive months or more [16]. Therefore, while treatment interruption can occur, it does not equate to LTFU under the WHO criteria.

Comparing our prevalence to other studies using the WHO definition of loss to follow-up, or some variation of it, a register-based retrospective analysis conducted at King Fahad Medical City in Riyadh, Kingdom of Saudi Arabia, from 2018 to 2023 to determine the factors related to unfavourable treatment outcomes involving 427 pTB patients reported only 4.6% loss to follow-up (defined as patients who did not start treatment or whose treatment was interrupted for two consecutive months or more) [17]. Another study, from continental Portugal, which focused on new pTB cases diagnosed between 2000 and 2013, reported an overall LTFU rate of 4.88%, with LTFU defined as being off drugs for more than 8 weeks after completing at least 1 month of treatment [18]. This rate is even lower than our observed prevalence, suggesting regional variations in adherence. A retrospective analysis from Georgia (2015–2017) on bacteriologically confirmed pulmonary TB patients reported a higher rate of 12% lost to follow-up (defined as treatment interruption for more than two consecutive months) [19], which is higher than results. These variations suggest variability in patient adherence challenges across countries and regions. However, they can also be influenced by differences in study methodology, including diagnostic criteria, follow-up protocols, data collection methods, and the specific healthcare settings involved.

Age was a significant factor in our study, with patients aged 25–44 years having the highest odds of LTFU (aOR 2.83), nearly three times that of patients older than 44 years. Similar patterns of association with regard to age of LTFU patients have been seen in studies in other countries [9,10,20], though strictly equal age group comparisons are not possible since age group categories differ between studies. In a Moroccan study, a patient age of less than 50 years was associated with LTFU (versus ≥ 50) [9], whereas in Karachi, Pakistan, patients aged 35 and above had higher LTFU compared to younger patients [20]. In a study in the Brazilian Amazon, in which age was analysed as a continuous variable, the odds of LTFU increased with age [10]. A plausible reason for the higher risk of LTFU among the working age group is that human productivity is often at its peak during this life stage, and consequently there are higher burdens of personal and work responsibilities, which may become barriers to adhering to treatment. One study that presented an exception was a South African study which found the highest rate of LTFU in the youngest age group (15–24 years) [21], and lower LTFU occurred among older patients, which the authors surmised to be due to older patients having better coping strategies during treatment.

No significant association was found for knowledge of tuberculosis per se, whereas other studies have shown that inadequate knowledge of TB is highly associated with increased odds of LTFU, such as a study by Muture et al. in Nairobi, Kenya (OR 8.67) [4]. However, patients’ level of education was a significant factor, although, surprisingly, knowledge of TB specifically was not significant. Patients who had an education level of secondary school or less had a more than three times higher rate of LTFU than their higher-educated counterparts (aOR 3.78). Other studies that examined the role of education as a factor, such as Garrido et al. in the Brazilian Amazon, reported similarly [10], though at a lower odds ratio (aOR 1.35) [22]. This finding is expected as a low level of education is also a factor for non-adherence to treatment for chronic non-infectious ailments [23]. Patients with lower levels of education may have limited health literacy, financial constraints, and time constraints due to having less flexible jobs and a lack of social support, making it harder for them to follow through appointments and treatments. Furthermore, patients with lower education levels may have different cultural beliefs or experiences that affect their attitudes towards medical treatment. However, interestingly, higher-educated patients had a higher risk of LTFU if they were unaware that the disease being treated was TB. The significant interaction between education level and TB awareness (*p* = 0.017) suggests that awareness modifies the effect of education on adherence. Notably, among patients with tertiary education, those who were unaware of their TB diagnosis had a higher probability of LTFU, whereas those with the same education level who were aware of their diagnosis were substantially less likely to be lost to follow-up. This finding suggests that higher education alone does not ensure better adherence unless paired with adequate awareness and understanding of the illness. In contrast, awareness appeared to have less impact among patients with lower educational levels, possibly due to limited health literacy or other socioeconomic barriers that persist regardless of awareness. It is possible that even highly educated patients may not completely understand their diagnosis. This could stem from complex medical terminology. Additionally, among the highly educated, the disease’s lower prevalence in their typical social or professional environments may reduce their spontaneous exposure to information about it. The prevailing perception of TB as a disease predominantly associated with poverty and marginalised communities can also lead higher-educated individuals to implicitly distance themselves from the diagnosis. A word of caution though, while a large proportion of higher-educated patients who were unaware of their diagnosis were LTFU (40%), it is important to acknowledge that this finding is based on an especially small sample size within this specific subgroup (*n* = 5).

With regard to marital status, there were two-fold higher odds of LTFU among patients who were not married (single, divorced, or widowed) (aOR 2.0). A study in Shenzhen city in the People’s Republic of China showed similar findings [24]. Suliman likewise found a significantly higher prevalence of TB treatment interruption among divorced patients [5], but not among single, widowed, or separated patients, compared to patients who were married. Spousal support has been reported to have a positive influence on adherence to treatment for various diseases [25]. This evidence strongly suggests that having a spouse has a positive influence on treatment adherence and reducing LTFU among TB patients as well. The spouse could provide support by reminding and encouraging the patient to continue with medications, and perhaps also in terms of logistically and financially supporting patients to adhere to the treatment.

The association between smoking and TB treatment LTFU found in other studies [5,26,27] is reinforced in this study, wherein smokers showed almost threefold higher odds of LTFU (aOR 2.77) compared to non-smokers. A study of smoking TB patients in Selangor found a 14.7% prevalence of LTFU [11]. Smokers have been documented to have lower compliance to recommended measures for other, albeit non-infectious, chronic diseases such as cancer screening and hypertension medication [28]. The authors speculated that this could be ascribed to the reasoning that since smoking is already unhealthy, it provides an excuse or justification for other unhealthy behaviours, which could be applied to smoking TB patients as well.

These findings highlight the need for targeted policy and programmatic interventions to improve TB treatment adherence, particularly among high-risk groups. The higher LTFU rate among individuals in the productive age group suggests that socioeconomic pressures may hinder treatment compliance. To address this, the Ministry of Human Resources and social security agencies could implement supportive measures such as paid medical leave, flexible work-from-home options, and financial assistance during the treatment period. Legal protections should also be considered to safeguard against job loss due to TB diagnosis or treatment.

Among single, divorced, or widowed individuals, who were also more likely to be lost to follow-up, healthcare facilities could establish peer support groups to offer both emotional and practical assistance throughout treatment. Given the well-established link between smoking and poor TB outcomes, smoking cessation support should be integrated into TB care, including counselling, nicotine replacement therapy, and behavioural interventions. The significant interaction between education level and TB awareness further underscores the need for tailored health communication strategies. The Ministry of Health could lead targeted public awareness campaigns, while the Ministry of Higher Education could incorporate mandatory TB education into tertiary enrolment processes.

In some countries, video direct observed therapy (vDOT) is being used as a digital alternative to traditional directly observed therapy (DOT), particularly for patients facing logistical or socioeconomic barriers [29]. It can also minimise barriers related to social stigma. This approach allows for flexible, remote monitoring of treatment while maintaining adherence oversight, and should be integrated into national TB control strategies with appropriate privacy safeguards and digital infrastructure support. Finally, legislative measures that promote treatment adherence, such as enforcement of the Prevention and Control of Infectious Diseases Act 1988 (Act 342) [30], should be actively implemented as part of TB control efforts. When applied with due consideration for patient rights and ethical standards, such laws can enhance accountability, reduce LTFU rates, and support the overall effectiveness of Malaysia’s national TB strategy.

This study has several limitations. Patients’ socioeconomic status (SES) was not examined, although it could be a strong predictor of patient LTFU. SES is a multifaceted construct that encompasses various factors such as income, occupational status, and education. Income data was collected but was not analysed due to the pervasiveness of missing values. Although occupation was captured, the wide range and diversity of responses limited the feasibility of analysis. As such, occupations were categorised into employed and unemployed. However, we did include education attainment in our analysis, which is one of the main indicators of SES, though it may not fully represent SES. Treatment LTFU was determined via various means, one of which was through cross-checking the TB case registry. In some cases, the data in the registry may not have been updated in a timely manner, possibly resulting in under-reporting of LTFU. While the knowledge and stigma scales used were adopted from a WHO Eastern Mediterranean study and have been applied across multiple countries, they were not formally validated in our local population. Although we conducted pre-testing to ensure clarity and comprehensibility (face validity), full psychometric validation such as construct validity or reliability testing was not performed. Therefore, the interpretation of results related to these scales should be made with caution, and future studies are encouraged to validate these instruments in the local context.

## 5. Conclusions

The demographic factors of age, marital status, and smoking status were associated with patient LTFU in TB treatment. Screening patients’ social background when initiating treatment, giving closer attention to smokers and single, divorced, or widowed patients, to minimise their potential to LTFU and ensuring that they have adequate social support systems, followed by provision of additional counselling and follow-up phone calls for instance, may help increase treatment adherence and reduce LTFU. We found that a lack of awareness about a TB diagnosis more strongly impacted adherence among higher-educated patients. This indicates that education alone does not guarantee understanding. To address this, healthcare providers should implement structured patient engagement strategies such as counselling, teach-back methods, and consistent follow-up communication to ensure that all patients, regardless of educational background, fully comprehend their diagnosis. Such approaches are essential to improving treatment adherence and reducing the risk of LTFU.

## Figures and Tables

**Table 1:** Proportion of loss to follow-up (LTFU) by sociodemographic variable (*n* = 699).

Variable	All Patients (*n* = 699)	Lost to Follow-Up (*n* = 55)	Chi-Square (*p*-Value)
	*n* (%) ^a^	*n* (%) ^b^	
Gender			2.87 (0.900)
Male	434 (62.1)	40 (9.2)	
Female	265 (37.9)	15 (5.7)	
Age			11.93 (0.008) **
18–24 (young adults)	119 (17.0)	9 (7.6)	
25–44 (adults)	274 (39.2)	32 (11.7)	
45–64 (middle age)	253 (36.2)	14 (5.5)	
65 and above (seniors)	53 (7.6)	0 (0.0)	
Ethnicity			7.00 (0.136)
Malay	404 (57.8)	30 (7.4)	
Chinese	71 (10.2)	5 (7.0)	
Indian	87 (12.4)	6 (6.9)	
Other indigenous peoples (Sabah/Sarawak/Orang Asli)	44 (6.3)	8 (18.2)	
Others	93 (13.3)	6 (6.5)	
Educational level			18.82 (<0.001) ***
No formal education	32 (4.6)	0 (0.0)	
Primary school	148 (21.2)	7 (4.7)	
Secondary school	355 (50.8)	43 (12.1)	
Tertiary	164 (23.5)	5 (3.0)	
Nationality			0.297 (0.586)
Malaysian	606 (86.7)	49 (8.1)	
Non-Malaysian	93 (13.3)	6 (6.5)	
Marital status			10.58 (0.001) **
Married	435 (62.2)	23 (5.3)	
Single/Divorced/Widowed	264 (37.8)	32 (12.1)	
Employment status			8.22 (0.004) **
Unemployed	192 (27.5)	6 (3.1)	
Employed	507 (72.5)	49 (9.7)	
Presence of comorbidities			0.001 (0.977)
Yes (≥1 comorbidity)	319 (45.6)	25 (7.8)	
No	380 (54.4)	30 (7.9)	

*p*-values from Pearson’s chi-square test unless specified; ^a^ column percentage; ^b^ row percentage, ** *p* value < 0.01; *** *p* value < 0.001.

**Table 2:** Proportion of loss to follow-up (LTFU) by lifestyle and psychosocial variable (*n* = 699).

Variable	All Patients (*n* = 699)	Lost to Follow-Up (*n* = 55)	Chi-Square (*p*-Value) ^c^
	*n* (%) ^a^	*n* (%) ^b^	
Ever smoked			12.22 (<0.001) ***
No	386 (55.2)	18 (4.7)	
Yes	313 (44.8)	37 (11.8)	
Alcohol drinker			0.45 (0.336) ^c^
No	673 (96.3)	52 (7.7)	
Yes	26 (3.7)	3 (11.5)	
Total delay in treatment			0.065 (0.798)
Low	357 (51.1)	29 (8.1)	
High	342 (48.9)	26 (7.6)	
Stigma score			3.32 (0.068)
Low	362 (51.8)	22 (6.1)	
High	337 (48.2)	33 (9.8)	
TB knowledge score			1.40 (0.237) ^d^
0	7 (1.0)	2 (28.6)	
1	7 (1.0)	0 (0)	
2	12 (1.8)	0 (0)	
3	50 (7.3)	4 (8.0)	
4	110 (16.1)	11 (10.0)	
5	202 (29.6)	19 (9.4)	
6	213 (31.2)	13 (6.1)	
7	81 (11.9)	5 (6.2)	
Aware of TB diagnosis			0.013 *^c^
Yes	656 (94.0)	47 (7.2)	
No	42 (6.0)	8 (19.0)	

*p*-values are from Pearson’s chi-square tests unless specified; ^a^ column percentage; ^b^ row percentage; ^c^ Fisher’s exact test; ^d^ linear-by-linear association; * *p* value < 0.0; *** *p* value <0.001.

**Table 3:** Proportion of loss to follow-up (LTFU) by treatment experience variable (*n* = 699).

Variable	All Patients (*n* = 699)	Lost to Follow-Up (*n* = 55)	Chi-Square (*p*-Value)
	*n* (%) ^a^	*n* (%) ^b^	
TB drug side effects			
Nausea	157 (22.5)	11 (7.0)	0.213 (0.645)
Abdominal pain	80 (11.5)	9 (11.3)	1.414 (0.234)
Headache	167 (23.9)	11 (6.6)	0.505 (0.477)
Loss of appetite	145 (20.8)	9 (6.2)	0.706 (0.401)
Jaundice	20 (2.9)	2 (10.0)	0.128 (0.721)
Numbness/Tingling	83 (11.9)	7 (8.4)	0.040 (0.842)
Rash	245 (35.1)	22 (9.0)	0.629 (0.428)
Fatigue	174 (24.9)	14 (8.0)	0.009 (0.925)
Joint pain	174 (24.9)	19 (10.9)	2.951 (0.086)
Visual changes	70 (10.0)	5 (7.1)	0.058 (0.809)
Hearing change	37 (5.3)	2 (5.4)	0.760 ^c^
Others	168 (24.1)	16 (9.5)	0.824 (0.364)
Cumulative side effects	548 (78.4)	46 (8.4)	0.967 (0.325)
Healthcare provider explained regarding:			
TB treatment regime	630 (91.0)	49 (7.8)	1.000 ^c^
Treatment duration	638 (92.1)	48 (7.5)	0.426 ^c^
Treatment side effects	570 (82.3)	42 (7.4)	0.803 (0.370)
Prognosis	622 (90.0)	49 (7.9)	0.034 (0.853)
Satisfaction with healthcare services			
Clinic hours are convenient	667 (96.0)	55 (8.2)	0.157 ^c^
Waiting time is reasonable (<1 h)	574 (82.5)	47 (8.2)	0.368 (0.544)
Healthcare workers treat me with respect	694 (99.6)	55 (7.9)	1.000 ^c^
Healthcare workers show a good attitude towards me	691 (99.3)	55 (8.0)	1.000 ^c^
I trust the healthcare workers	694 (99.6)	55 (7.9)	1.000 ^c^
I am currently feeling better	677 (97.1)	53 (7.8)	0.667 ^c^
I am satisfied with the services provided	688 (98.9)	55 (8.0)	1.000 ^c^
I am currently taking herbal medication/supplements	153 (22.0)	7 (4.6)	2.965 (0.085)

*p*-values from Pearson’s chi-square test unless specified; ^a^ column percentage; ^b^ row percentage; ^c^ Fisher’s exact test.

**Table 4:** Factors associated with TB treatment loss to follow-up (LTFU) (*n* = 698).

Factor	*p*-Value	aOR	95% CI
Age (years)			
18–24 (young adults)	0.249	1.79	0.66, 4.86
25–44 (adults)	0.004	2.83	1.40, 5.72
45 and above (middle age and older)		Ref.	
Marital status			
Single/divorced/widowed	0.019	2.17	1.14, 4.14
Married		Ref.	
Education			
Secondary or less	0.003	6.13	1.83, 20.53
Tertiary		Ref.	
Ever smoked			
Yes	0.002	2.65	1.43, 4.92
No		Ref.	
Ignorant of TB diagnosis			
Yes	0.001	38.14	4.10, 354.89
No		Ref.	
Education level × ignorant of TB diagnosis *	0.017	0.05	0.004, 0.580

* Interaction between education level and being ignorant of TB diagnosis. Hosmer–Lemeshow *p* = 0.746, overall correctly classified = 92.3%. The predictive model area under the ROC curve (76.1%) indicated acceptable model fit. No multicollinearity between predictors (variation inflation factor (VIF): <2.

## Data Availability

The data from this study is deposited in the Malaysian Ministry of Health’s National Institutes of Health data repository (NIH-DARS) at https://nihdars.nih.gov.my and may be available upon request to the repository administrators.

## Supplementary Materials

The following supporting information can be downloaded at: https://www.mdpi.com/article/10.3390/tropicalmed10080226/s1, Study questionnaire. The Supporting Information is available free of charge.

## Abbreviations

The following abbreviations are used in this manuscript:

LTFU	Loss to Follow-Up
pTB	Pulmonary tuberculosis
SES	Socioeconomic status
TB	Tuberculosis
