# Supplementary material for: Unmasking the Determinants of Loss to Follow-Up in Pulmonary Tuberculosis: A Study in Selangor, Malaysia
Source: Trop Med Infect Dis. 2025 Aug 12;10(8):226. doi: 10.3390/tropicalmed10080226 (PMC12390508; doi:10.3390/tropicalmed10080226)
Supplement: Supplementary file 1 [file tropicalmed-10-00226-s001.zip › tropicalmed-3670672-Supplementary file - Study questionnaire.pdf]

A1 Interviewer: __________ A2 Date of interview: ________________

DISTRICT & CLINIC CODES

District	*Dist. Code*	*KK*	*KK Code*
**H.Selangor**	01	Hulu Yam Baru	01
		Rasa	02
		Serendah	03
		Sg Selisek	04
		Kalumpang	54
		Kg. Soeharto	55
		HKKB	82
**K.Selangor**	02	Bestari Jaya	05
		Bukit Cherakah	06
		Jeram	07
		Kuala Selangor	08
		Sg Tengi Kanan	09
		Ijok	56
		Tg. Karang	57
		HTK	79
**Gombak**	03	AU2 Keramat	10
		Batu Arang	11
		Kuang	12
		Rawang	13
		Selayang Baru	14
		Taman Ehsan	15
		Ulu Klang	16
		HOAG	88
		HSEL	86
		HSgBlh	85
**H.Langat**	04	Balakong	17
		Bandar Seri Putra	18
		BB Bangi	19
		Beranang	20
		Bt 14 Cheras	21
		Bt 9 Cheras	22
		Kajang	23
		Sg Chua	24
		Hamp	84
		GSdg	77
		HKjg	83

District	*Dist. Code*	*KK*	*KK Code*
**Sepang**	05	Dengkil	25
		Salak	26
		Sepang	27
		Sg. Pelek	64
**Petaling**	06	Bt 13 Puchong	28
		Kelana Jaya	29
		Lembah Subang	30
		Paya Jaras	31
		Sek 7 Shah Alam	32
		Seri Kembangan	33
		HSA	87
**S.Bernam**	07	Bagan Terap	34
		Merbau Berdarah	35
		Sabak Bernam	36
		Sekinchan	37
		Sg Air Tawar	38
		Simpang Lima	76
		HTAJ	78
**K.Langat**	08	Jenjarom	39
		Kanchong Darat	40
		Kg Bandar	41
		Sg Lang Tengah	42
		Sijangkang	43
		T.Panglima Garang	44
		Tg Sepat	45
		HBAN	80
**Klang**	09	Anika	46
		Botanik	47
		Bukit Kuda	48
		Bukit Naga	49
		Kapar	50
		Pelabuhan Klang	51
		Pulau Indah	52
		Rantau Panjang	53
		HTAR	81

Part B. Sociodemographic characteristics

B1 Name of patient: ____

B2 NRIC: ☐☐☐☐☐☐ - ☐☐ - ☐☐☐☐
or
Passport /Police/Army No.: ______________________________

B3 Sex:
Male ☐
Female ☐

Telefon: ______________________________________

B4 Date of birth: ____/____/______ (dd/mm/yyyy)

B5 Citizenship/Kewarganegaraan:
Malaysian citizen ☐ If Yes, go to B7
Permanent resident ☐
Non-Malaysian citizen ☐

5.2 How long have you stayed in Malaysia?___ years ___ months

B6 If non-Malaysian, specify nationality/

Indonesia	☐	Vietnam	☐
Myanmar	☐	China	☐
Bangladesh	☐	Pakistan	☐
Nepal	☐	Thailand	☐
India	☐	Others	☐ Specify:________________

B7 Ethnicity: (for Malaysians only)
Malay ☐
Chinese ☐
Indian ☐
Bumiputra Sabah/Sarawak ☐
Orang Asli ☐
Others ☐ Please specify:___________

B8 Education level
No formal education ☐
Primary school ☐
Secondary school ☐
Tertiary ☐

B9 Marital status
Single ☐ Married ☐ Divorced ☐ Widowed ☐

B10 Employment status (Before diagnosis)

Employed ☐ Describe occupation : ________________________

Unemployed ☐

B11 Employment status (Current)

Employed ☐ Describe occupation : ________________________
Bekerja

Unemployed ☐
Tidak bekerja

B12 Current residential address: ____

Postcode:_________Town/City___________ State__________

B13 Type of house *Jenis rumah*:

Flat	☐	Squatterhouse/	☐
Hostel	☐	*Rumah kongsi*	☐
Apartment/Condominium	☐	Shophouse	☐
Terrace house/Bungalow	☐	Homeless	☐

B14 Number of households in the same house
(Note: A household refers to a household unit in which members share the same kitchen)

Household 1→ Household size: _____ persons
Households 2→ Household size: _____ persons
Households 3→ Household size: _____ persons
Households 4→ Household size: _____ persons

B15 Monthly personal income: RM _______

B16 Monthly household income: RM _______
(Sum total of income contributed by all members of the household)

Part C. Lifestyle variables

C1 Smoking status

Never smoked ☐ Current smoker ☐ Former smoker ☐

C2 **If current smoker**, specify number of cigarettes smoked in a typical day: ______ sticks

C3 **If current smoker** or **former smoker**, duration of smoking: _____years _____months

C4 In the past 7 days, did you consume alcohol?

Yes ☐
No ☐

Part D. Knowledge & Stigma

D1 Have you previously heard of TB?

- Yes ☐
- No ☐

D2 Source of information on TB

- Health staff ☐
- Pamphlets / *Brochure* ☐
- School education ☐
- Health talk ☐
- Health magazine ☐
- TV documentary ☐
- Friends/Relatives ☐
- Social media (Facebook, Whatsapp,etc) ☐
- TB disease in friends/relatives ☐
- Others.- Specify:__________ ☐

D3 Correctness of information on TB

		Patient's answer	Interviewer assessment		
			Correct	Incorrect	Don't know
D3.1	What kind of disease do you have?	______________	☐	☐	☐
D3.2	Is TB hereditary?	Yes ☐ No ☐	☐	☐	☐
D3.3	Is TB contagious?	Yes ☐ No ☐	☐	☐	☐
D3.4	Is TB curable?	Yes ☐ No ☐	☐	☐	☐
D3.5	Do you know if there is a vaccine for TB?	Yes ☐ No ☐	☐	☐	☐
D3.6	Do you know the approximate duration of treatment?	______________	☐	☐	☐
D3.7	Do you know the kind of TB drugs?	1)__________________<br>2)__________________<br>3)__________________<br>4)__________________	☐	☐	☐

D4 TB Stigma

		Strongly agree	Agree	Average	Do not agree	Do not agree at all
D4.1	Do you feel ashamed for having TB?	①	②	③	④	⑤
D4.2	Do you have to hide your TB diagnosis from other people?	①	②	③	④	⑤
D4.3	Does TB affect your relationship with others?	①	②	③	④	⑤
D4.4	Is TB very costly due to the long duration of the disease?	①	②	③	④	⑤
D4.5	Do you prefer to live isolated since you got TB diagnosis?	①	②	③	④	⑤
D4.6	Does TB affect your work performance?	①	②	③	④	⑤
D4.7	Does TB affect marital relations?	①	②	③	④	⑤
D4.8	Does TB affect family responsibilities?	①	②	③	④	⑤
D4.9	Do you think there is less chances of marriage due to TB diagnosis?	①	②	③	④	⑤
D4.10	Does TB affect your family relations?	①	②	③	④	⑤
D4.11	Does TB cause female infertility?	①	②	③	④	⑤
D4.12	Does TB lead to serious complications during pregnancy?	①	②	③	④	⑤
D4.13	Does TB affect breastfeeding?	①	②	③	④	⑤
D4.14	Does TB affect pregnancy outcome?	①	②	③	④	⑤
D4.15	Is a girl unable to decide for getting TB treatment?	①	②	③	④	⑤

Part E. Laboratory & Clinical variables

E1 Date of TB diagnosis: ____/____/______ (dd mm yyyy)   E2 Multidrug resistant (MDR-TB)? Yes ☐ No ☐

E3 Laboratory investigation of TB:

Investigation	Number of times	Health facility* of each facility where test was performed	Date performed	Date official results received	Result (✓ where applicable)			Not performed
					Positive	Negative	Inconclusive	
E3.1 Chest X-ray		☐<br>☐<br>☐	__/__/____<br>__/__/____<br>__/__/____	__/__/____<br>__/__/____<br>__/__/____	☐<br>☐<br>☐	☐<br>☐<br>☐	☐<br>☐<br>☐	☐
E3.2 Sputum smear		☐<br>☐<br>☐	__/__/____<br>__/__/____<br>__/__/____	__/__/____<br>__/__/____<br>__/__/____	☐<br>☐<br>☐	☐<br>☐<br>☐	☐<br>☐<br>☐	☐
E3.3 Sputum culture		☐<br>☐<br>☐	__/__/____<br>__/__/____<br>__/__/____	__/__/____<br>__/__/____<br>__/__/____	☐<br>☐<br>☐	☐<br>☐<br>☐	☐<br>☐<br>☐	☐
E3.4 GeneXpert			__/__/____	__/__/____	☐	☐	☐	☐
E3.5 Others. Specify: ________________		☐	__/__/____	__/__/____	☐	☐	☐	☐

* 1 – GP
2 – Private hospital
3 – Klinik kesihatan/Klinik desa/Klinik 1 Malaysia
4 – Government hospital

E4 Do you have any of the following conditions:

HIV/AIDS	☐	
Diabetes mellitus	☐	
Asthma	☐	
COPD	☐	
Cancer	☐	
Autoimmune (SLE, etc.)	☐	
Others	☐	Specify:______________ _

E5 Anthropometric measurements: (From review of records)

Weight: _____ kg

Height: ______ cm

E6 Do you know anyone who is known to have TB?

Yes ☐

No ☐

E7	If Yes, (had contact with someone known to have TB)		Year had contact
E7.1	Immediate family member/	☐	_______
E7.2	Relatives/	☐	_______
E7.3	Work colleague	☐	_______
E7.4	Friend/s	☐	_______
E7.5	Neighbour	☐	_______
E7.6	Others. Specify: ________________	☐	_______
E7.7	Does not know anyone with TB/	☐	

Part F. Chronology of symptom onset & treatment

F1 Chief symptoms and date of onset:

Symptom		Yes	If **Yes**,<br>Please specify onset date<br>*dd/mm/yyyy*
F1.1	Cough (>2 weeks with or w/o sputum)	☐	____/____/________
F1.2	Fever	☐	____/____/________
F1.3	Night sweats	☐	____/____/________
F1.4	Loss of appetite	☐	____/____/________
F1.5	Unexplained loss of weight	☐	____/____/________
F1.6	Haemoptysis (Coughing up blood)	☐	____/____/________
F1.7	Pleuritic chest pain	☐	____/____/________
F1.8	Others. Specify:__________________	☐	____/____/________

F2 What did you do after onset of the symptoms?

Seek treatment from healthcare practitioner /	☐
Self-medicate (take herbs, supplements, OTC drugs) /	☐
Traditional medicine practitioner (Sinseh, tabib) /	☐
Seek medication from pharmacy /	☐
Others. Please specify:____________	☐

F3 Which symptom/s made you seek healthcare? *(Multiple answers are allowed)*

Symptom	Yes
Cough	☐
Fever	☐
Night sweats	☐
Loss of appetite	☐
Loss of weight	☐
Haemoptysis	☐
Pleuritic chest pain	☐
Others Specify:______________	☐

F4 Facility where you sought consultation before commencing TB treatment (in chronological order):

1- GP / *Klinik swasta*
2- Private hospital / *Hospital swasta*
3- Klinik kesihatan/Klinik Desa/Klinik 1 Malaysia
4- Government hospital / *Hospital kerajaan*
5- TCM practitioner / *Pengamal perubatan tradisional/alternatif*

Order	Facility where you sought consultation *Tempat mendapat rawatan*	Date *Tarikh*	Was TB diagnosis made here? *Adakah diagnosa TB dibuat di sini?* Yes/No	Date *Tarikh diagnosa*	Was TB treatment started? *Adakah rawatan TB dimulakan?* Yes/No	Date *Tarikh rawatan dimulakan*
1st						
2nd						
3rd						
4th						
5th						

F5 Duration from onset of <u>first</u> symptom to <u>first</u> medical consultation? *Tempoh masa antara simptom muncul dan berjumpa doktor perubatan?*

< 2 weeks ☐
Kurang dari 2 minggu
≥ 2 weeks ☐
2 minggu ke atas

If duration from onset of <u>first</u> symptom to <u>first</u> medical consultation >2 weeks:

F6 Why did you delay making a visit to a medical practitioner?

- Fear of what would be found on diagnosis ☐
- Hoped symptoms would disappear by itself ☐
- Fear of social isolation ☐
- Financial constraints ☐
- Inadequate staff attitude ☐
- Poor quality of health services / ☐
- Others ☐ Please specify :_______________
- No delay (<2 weeks) ☐

If, first action after onset of symptoms is → consulted a <u>healthcare provider</u>:

F7 What are the reasons?

(Multiple answers allowed)

- Accessible / *Mudah berjumpa doktor* ☐
- Confidence in getting cured / *Yakin boleh sembuh* ☐
- Services available anytime / *Perkhidmatan boleh didapati pada bila-bila masa* ☐
- Referred by previous health service / *Dirujuk oleh klinik* ☐
- Free services / *Perkhidmatan percuma* ☐
- Advised by somebody / *Dinasihatkan oleh orang lain* ☐
- Others / *Lain-lain* ☐ Please specify:____________

If first action after onset of symptoms is → <u>did not</u> consult healthcare provider:

F8 Why did you <u>not consult</u> a health facility despite onset of symptoms?

(Multiple answers allowed

- F8.1 Too far / *Terlalu jauh* ☐
- F8.2 Long waiting time / *Masa menunggu terlalu lama* ☐
- F8.3 Costly / *Terlalu mahal* ☐
- F8.4 Bad experience (Dissatisfied with the service) / *Pengalaman pahit (Tidak berpuashati dengan layanan)* ☐
- F8.5 Others / *Lain-lain* ☐ Please specify:___________________

F9 Distance from home to the nearest healthcare facility ☐☐ km
Jarak dari rumah ke klinik/pusat kesihatan yang terdekat

F10 Time to reach home from the nearest healthcare facility ☐ hours ☐ minutes

Part G. Default variables

(To be filled at follow-up interview more than 2 weeks after treatment initiation)

G1 Have you experienced any of the following side effects?

	Yes	No		Yes	No
Nausea	☐	☐	Rash	☐	☐
Abdominal pain	☐	☐	Fatigue	☐	☐
Headache	☐	☐	Joint pain	☐	☐
Loss of appetite	☐	☐	Visual change	☐	☐
Jaundice/Yellow color	☐	☐	Hearing change	☐	☐
Numbness/Tingling	☐	☐	Other	☐	☐

G2 Did the healthcare provider explain to you about the following:

		Yes	No
4.1	TB treatment regime	☐	☐
4.2	Duration of treatment	☐	☐
4.3	Side effects	☐	☐
4.4	Expected development of a disease or of the chances of getting better (Prognosis)	☐	☐

G3 Support from the healthcare provider:

		Yes	No
5.1	Are clinic hours convenient?<br>Adakah waktu klinik dibuka sesuai?	☐	☐
5.2	Is the waiting time reasonable (<1 hour)?<br>Adakah tempoh menunggu giliran munasabah (<1jam)?	☐	☐
5.3	Did the healthcare worker treat you with respect?<br>Adakah kakitangan kesihatan menghormati anda?	☐	☐
5.4	Did the healthcare worker have good attitude towards you?<br>Adakah kakitangan kesihatan menunjukkan sikap yang baik?	☐	☐
5.5	Do you trust the healthcare worker?<br>Adakah anda menaruh kepercayaan terhadap kakitangan kesihatan di sini?	☐	☐

G4 Are you satisfied with the services provided?
Adakah anda berpuashati dengan perkhidmatan yang diberi?

Yes ☐
No ☐

G5 Are you feeling better?
Adakah anda berasa lebih sihat?

Yes ☐
No ☐

G6 Are you currently taking any herbal medication/supplements?
Adakah anda mengambil sebarang makanan tambahan atau ubat-ubatan tradisional?

Yes ☐
No ☐

Part H. Follow-up

H1 Date of initiation of intensive phase: ____/____/______ (dd/mm/yyyy)

H2 Date of initiation of maintenance phase: ____/____/______ (dd/mm/yyyy)

Day	DOT	Self	Day	DOT	Self	Day	DOT	Self	Day	DOT	Self	Day	DOT	Self	Day	DOT	Self	Day	DOT	Self	Day	DOT	Self
1	☐	☐	1	☐	☐	1	☐	☐	1	☐	☐	1	☐	☐	1	☐	☐	1	☐	☐	1	☐	☐
2	☐	☐	2	☐	☐	2	☐	☐	2	☐	☐	2	☐	☐	2	☐	☐	2	☐	☐	2	☐	☐
3	☐	☐	3	☐	☐	3	☐	☐	3	☐	☐	3	☐	☐	3	☐	☐	3	☐	☐	3	☐	☐
4	☐	☐	4	☐	☐	4	☐	☐	4	☐	☐	4	☐	☐	4	☐	☐	4	☐	☐	4	☐	☐
5	☐	☐	5	☐	☐	5	☐	☐	5	☐	☐	5	☐	☐	5	☐	☐	5	☐	☐	5	☐	☐
6	☐	☐	6	☐	☐	6	☐	☐	6	☐	☐	6	☐	☐	6	☐	☐	6	☐	☐	6	☐	☐
7	☐	☐	7	☐	☐	7	☐	☐	7	☐	☐	7	☐	☐	7	☐	☐	7	☐	☐	7	☐	☐
8	☐	☐	8	☐	☐	8	☐	☐	8	☐	☐	8	☐	☐	8	☐	☐	8	☐	☐	8	☐	☐
9	☐	☐	9	☐	☐	9	☐	☐	9	☐	☐	9	☐	☐	9	☐	☐	9	☐	☐	9	☐	☐
10	☐	☐	10	☐	☐	10	☐	☐	10	☐	☐	10	☐	☐	10	☐	☐	10	☐	☐	10	☐	☐
11	☐	☐	11	☐	☐	11	☐	☐	11	☐	☐	11	☐	☐	11	☐	☐	11	☐	☐	11	☐	☐
12	☐	☐	12	☐	☐	12	☐	☐	12	☐	☐	12	☐	☐	12	☐	☐	12	☐	☐	12	☐	☐
13	☐	☐	13	☐	☐	13	☐	☐	13	☐	☐	13	☐	☐	13	☐	☐	13	☐	☐	13	☐	☐
14	☐	☐	14	☐	☐	14	☐	☐	14	☐	☐	14	☐	☐	14	☐	☐	14	☐	☐	14	☐	☐
15	☐	☐	15	☐	☐	15	☐	☐	15	☐	☐	15	☐	☐	15	☐	☐	15	☐	☐	15	☐	☐
16	☐	☐	16	☐	☐	16	☐	☐	16	☐	☐	16	☐	☐	16	☐	☐	16	☐	☐	16	☐	☐
17	☐	☐	17	☐	☐	17	☐	☐	17	☐	☐	17	☐	☐	17	☐	☐	17	☐	☐	17	☐	☐
18	☐	☐	18	☐	☐	18	☐	☐	18	☐	☐	18	☐	☐	18	☐	☐	18	☐	☐	18	☐	☐
19	☐	☐	19	☐	☐	19	☐	☐	19	☐	☐	19	☐	☐	19	☐	☐	19	☐	☐	19	☐	☐
20	☐	☐	20	☐	☐	20	☐	☐	20	☐	☐	20	☐	☐	20	☐	☐	20	☐	☐	20	☐	☐
21	☐	☐	21	☐	☐	21	☐	☐	21	☐	☐	21	☐	☐	21	☐	☐	21	☐	☐	21	☐	☐
22	☐	☐	22	☐	☐	22	☐	☐	22	☐	☐	22	☐	☐	22	☐	☐	22	☐	☐	22	☐	☐
23	☐	☐	23	☐	☐	23	☐	☐	23	☐	☐	23	☐	☐	23	☐	☐	23	☐	☐	23	☐	☐
24	☐	☐	24	☐	☐	24	☐	☐	24	☐	☐	24	☐	☐	24	☐	☐	24	☐	☐	24	☐	☐
25	☐	☐	25	☐	☐	25	☐	☐	25	☐	☐	25	☐	☐	25	☐	☐	25	☐	☐	25	☐	☐
26	☐	☐	26	☐	☐	26	☐	☐	26	☐	☐	26	☐	☐	26	☐	☐	26	☐	☐	26	☐	☐
27	☐	☐	27	☐	☐	27	☐	☐	27	☐	☐	27	☐	☐	27	☐	☐	27	☐	☐	27	☐	☐
28	☐	☐	28	☐	☐	28	☐	☐	28	☐	☐	28	☐	☐	28	☐	☐	28	☐	☐	28	☐	☐
29	☐	☐	29	☐	☐	29	☐	☐	29	☐	☐	29	☐	☐	29	☐	☐	29	☐	☐	29	☐	☐
30	☐	☐	30	☐	☐	30	☐	☐	30	☐	☐	30	☐	☐	30	☐	☐	30	☐	☐	30	☐	☐
31	☐	☐	31	☐	☐	31	☐	☐	31	☐	☐	31	☐	☐	31	☐	☐	31	☐	☐	31	☐	☐
